# Difficulties in tuberculosis infection control in a general hospital of Vietnam: a knowledge, attitude, and practice survey and screening for latent tuberculosis infection among health professionals

**DOI:** 10.1186/s12879-019-4593-z

**Published:** 2019-11-08

**Authors:** Chau Quy Ngo, Toshie Manabe, Giap Van Vu, Hanh Thi Chu, Trang Thi Thu Vu, Trang Thu Tran, Lan Thi Phuong Doan, Jin Takasaki, Koichiro Kudo

**Affiliations:** 1 Bach Mai Hospital, Hanoi, Vietnam; 2 Jichi Medical University, Center for Community Medicine, 3311-1 Yakushiji, Shimotsuke, Tochigi, 329-0498 Japan; 3 National Center for Global Health and Medicine, Division of Pulmonary Medicine, Tokyo, Japan; 4 Waseda University Organization for Regional and Inter-Regional Studies, Tokyo, Japan; 5 Yurin Hospital, Tokyo, Japan

**Keywords:** Tuberculosis, KAP, TB infection control, Latent tuberculosis infection, General hospital, Tuberculin skin test, N95 respirator

## Abstract

**Background:**

In Vietnam, a country with a high tuberculosis (TB) burden, health professionals in both TB-specialized and non-TB-specialized general hospitals have a high risk of acquiring TB. The aims of the present study were to clarify the difficulties in TB infection control at non-TB specialized hospitals and whether any associated risks of latent TB infection exist among health professionals in Vietnam.

**Methods:**

We conducted a cross-sectional study in a national tertiary and general hospital of Hanoi, Vietnam. Participants were health professionals, including physicians, nurses, and other health professionals. We assessed difficulties in TB infection control by conducting a knowledge, attitude, and practice (KAP) survey. We also collected data on the results of tuberculin skin tests (TSTs) conducted during health check-ups for hospital staff to determine whether health professionals had latent TB infection or TB disease. KAP scores were compared among health professional groups (physicians vs. nurses vs. other health professionals). Factors influencing knowledge scores were evaluated using multiple regression analysis.

**Results:**

A total 440 health professionals at the study site participated in the KAP survey, and we collected the results of TSTs from a total of 299 health professionals. We observed a high prevalence of latent TB infection (74.2%), especially among participants in the emergency department. Although participants had high KAP scores, some topics were less understood, such as symptoms and risks of TB, proper use of protective equipment such as N95 respirators, and preventing transmission by patients with confirmed or suspected TB. Factors influencing knowledge scores associated with TB were age, a belief that TB is the most important infectious disease, being a medical professional, having previously attended workshops or seminars, and knowing that Vietnam has a high burden of TB.

**Conclusion:**

In a non-TB specialized hospital of Vietnam, we observed a risk of TB infection among health professionals and difficulties in properly controlling TB infection. Early awareness regarding patients with suspected TB, to apply proper measures and prevent transmission, and education regarding obtaining updated knowledge through scientific information are crucial to enhancing TB infection control in general hospitals of Vietnam.

## Background

Tuberculosis (TB) remains an important global health burden, with an estimated 10 million new TB cases and 1.3 million TB-related deaths worldwide [1]. In Vietnam, the Global TB report 2018 estimated that there were 124,000 new TB cases and 12,000 TB-related deaths in 2017, placing the country in the category of a high TB-burden country [1]. Prevention and infection control in Vietnam remain serious challenges in reducing the TB prevalence and mortality.

Health professionals in hospitals are at increased risk for acquiring TB, which can lead to severe medical consequences [2, 3]. In particular, health professionals at non-TB-specialized hospitals in low- and middle-income countries may face difficulties in preventing nosocomial TB infection, which may cause latent TB infection [4, 5]. Such difficulties are owing to lower awareness of TB infection among both patients and health professionals as well as insufficient TB prevention measures in hospitals and limited human and medical resources. Furthermore, prior to receiving a diagnosis of TB, a high proportion of patients with TB visit the emergency department and outpatient clinics that treat high-risk diseases [6]. Although guidelines for TB infection control are available [7, 8], implementation must be modified according to the health care setting, such as whether the hospital has the role of making an initial diagnosis (non-TB-specialized general hospitals) or receiving patients already diagnosed with TB (TB-specialized hospitals).

A knowledge, attitude, practice (KAP) survey is used to assess a person’s understanding, related thoughts and beliefs, and skills. KAP assessments can also provide useful data regarding deficits and gaps in TB control measures, which can help in focusing on subsequent infection control strategies [9]. Given the importance of reducing nosocomial TB transmission, the World Health Organization (WHO) recommends use of a KAP survey as a valuable tool when considering TB control strategies [10]. Several studies from high TB-burden countries have previously reported the results of KAP surveys conducted among health professionals in terms of TB control and treatment strategies [11–14]. However, according to our understanding, difficulties in TB infection control at tertiary general hospitals in Vietnam have not been comprehensively assessed, including investigation of difficulties among health professionals, problems with facilities, and the epidemiology of latent TB infection among health professionals.

The aim of the present study was thus to clarify whether health professionals at a general hospital in a high TB-burden country (Vietnam) have appropriate KAP related to TB infection control. Screening for *Mycobacterium tuberculosis* infection using the tuberculin skin test (TST) [15] was also performed among health professionals, to identify any existing factors associated with latent TB infection. The results of this study will contribute to improving the health status of the population in Vietnam including the TB infection control.

## Methods

### Study structure

The present study consisted of two parts: 1) a KAP survey conducted among health professionals, to identify measures taken in TB infection control at a general hospital in Vietnam, a high TB-burden country; 2) TSTs performed among these health professionals to determine their KAP related to the infection of *M. tuberculosis*, as the examination for latent TB infection or TB disease.

This study was approved by the institutional review board of National Bach Mai Hospital (BMH). Written informed consent was obtained from all study participants.

### Study site and participants

A cross-sectional study was conducted at BMH in Hanoi, Vietnam, a tertiary general hospital designated as the central hospital by the Ministry of Health of Vietnam [16, 17]. In the Vietnamese health care system, treatment and hospitalization of patients with TB are carried out in TB-specialized hospitals. Only the initial diagnosis of TB is made at non-TB-specialized general hospitals, and patients are immediately transferred to a TB-specialized hospital if a diagnosis of TB is received. However, the central hospital is responsible for attending patients who are unable to be adequately treated in local hospital settings, such as provincial and district hospitals. Therefore, BMH was selected as the site for the present study as a high-risk medical organization, despite being a non-TB specialized hospital.

Among 3165 employees in the BMH, 594 full-time health professionals (physicians, nurses, other health professionals, and medical clerks) work in departments with high TB risk including respiratory centers; the emergency; the infectious disease, nephrology, hematology, and endocrinology departments, and outpatient clinics. These health professionals were selected as the study participants.

### KAP survey methodology

We carried out the questionnaire survey in June 2018. A self-administered, structured questionnaire was originally designed by the study investigators based on “A guide to developing knowledge, attitude, and practice surveys” by the WHO [10] and according to the published literature. The questionnaire was designed to assess knowledge, attitudes, and practices related to TB infection and disease. To identify the occupational risk of TB infection, the questionnaire was also designed to collect the following information: demographics, time working in the current position, work experience in departments with high TB risk, TB history, TB history of family members, comorbidities, and Bacillus Calmette–Guerin (BCG) vaccination history. The English version of the survey, comprising 53 questions, was translated into Vietnamese, then back-translated into English for validation. All questions were multiple choice or closed-ended, and participants chose their responses from a provided set of answers (Yes/No, True/False, or Agree/Disagree/Undecided).

The KAP survey was conducted on 2 days in June 2018. The study investigators visited each department in BMH during working hours and collected the self-administered questionnaires from participants, to avoid the exchange of information among participants in a single medical facility.

### Screening for *M. tuberculosis* infection among participants

We collected data of TSTs from the results of *M. tuberculosis* infection screening performed during health check-ups for hospital staff, which were conducted during the same month with the KAP survey in the present cross-sectional study. We defined *M. tuberculosis* infection as a persistent immune response to stimulation with *M. tuberculosis* antigens, as indicated by the results of a TST [15].

In the present study, TST results were determined according to classification of the TST reaction of the Center for Disease Control and Prevention. A TST result was considered positive when any of the following conditions were met: 1) induration of ≥5 mm in participants who had recent contact with a person with TB disease; 2) induration of ≥10 mm in participants, in the absence of recent contact with pulmonary or laryngeal TB in the absence of BCG vaccination; or 3) induration of ≥15 mm in BCG-vaccinated participants with no other previous considerations [15]. The TST was considered to be negative for indurations smaller than the aforementioned diameters [15].

### Statistical analysis

Most variables derived from questions of KAP survey were categorical, with the exception of age, years of working experience, and KAP scores. Categorical variables are summarized as percentages, and continuous variables are presented as the median and interquartile range (IQR; 25–75%) or mean and standard deviation (SD). Each variable was compared among medical professional groups using the χ^2^ test or Fisher’s exact test for categorical variables and the Mann–Whitney *U* test or Kruskal–Wallis test for continuous variables.

On the KAP survey, each question was scored as follows, using a 3-point Likert-type scale: 3 points were assigned for responses of “agree,” which represented a positive attitude; 2 points were assigned for “undecided”; and 1 point was assigned for “disagree,” KAP scores were calculated in accordance with participants’ responses using factor analysis and adjusted to yield a total score of 10. The KAP scores were then compared among medical professional groups.

To determine possible factors influencing the knowledge score, a step-wise selection method was used to select variables for multiple regression analysis with variables of general characteristics as well as variables if *p* value was < 0.05 by univariate analysis.

Statistical analyses were performed using IBM SPSS Statistics version 25.0 (IBM Corp., Armonk, NY, USA). In all analyses, significance levels were two-tailed, and *p* values *<*0.05 were considered statistically significant.

## Results

### General background of participants in the KAP survey

Of 594 health professionals in the targeted departments at the study site, a total of 440 (74.1%) health professionals participated in the questionnaire survey: 31.8% were physicians, 58.2% were nurses, and 10.0% were other health professionals, including laboratory technicians and medical clerks. Participant characteristics are shown in Table 1. Although the median age was significantly higher among physicians, the most frequent age in all three health professional groups was 30–39 years. The median years of working experience were similar among the three groups.

**Table 1:** Characteristics of participants according to health professionals

	Physician	Nurse	Others	*P* value
	*n* = 140	*n* = 256	*n* = 44	
Demographics				
Age-mean (SD), yr.	36.9 (8.1)	32.1 (6.9)	32.9 (6.9)	<0.001^‡^
≤ 29	30 (21.4)	96 (37.5)	14 (31.8)	
30–39	66 (47.1)	122 (47.7)	22 (50.0)	
≥ 40	44 (31.4)	38 (14.8)	8 (18.2)	
Gender-male, n (%)	2 (44.3)	42 (16.4)	14 (31.8)	<0.001^*^
Working conditions				
Working experience as your profession-median (IQR), yr.	9 (3–15)	6 (3–19)	8 (3–12)	0.131^§^
Work in high-risk position for TB, n (%)	103 (73.6)	194 (77.0)	32 (72.7)	0.678^*^
History of Education on TB, n (%)				
Ever attended any of educational programs regarding TB	80 (58.4)	62 (24.7)	7 (15.9)	<0.001^*^
History of TB disease, n (%)				
Past history	9 (6.5)	16 (6.3)	0 (0.0)	0.483^†^
Currently on TB treatment	1 (0.7)	5 (2.0)	0 (0.0)	0.322^†^
Information resources regarding TB, n (%) (*n* = 432)				
Seminar	64 (45.7)	34 (13.7)	6 (13.6)	<0.001^*^
Scientific research paper	60 (42.9)	22 (8.9)	7 (15.9)	<0.001^*^
Guidelines	78 (55.7)	89 (35.9)	16 (36.4)	<0.001^*^
University faculties	98 (70.0)	74 (29.8)	13 (29.5)	<0.001^*^
Friends and Colleagues	66 (47.1)	143 (57.7)	15 (34.1)	0.006^*^
Media news	70 (50.0)	184 (74.2)	28 (63.6)	<0.001^*^
Others	7 (5.0)	35 (14.1)	7 (15.9)	0.015^*^

*SD* Standard deviation, *IQR* Interquartile range, *TB* Tuberculosis

* χ2 test; †Fisher’s exact test; ‡Mann-Whitney U test; §Kruskal-Wallis test

Approximately 6.5% of physicians and 6.3% of nurses had a previous history of TB disease, and one physician and five nurses were currently receiving medication for TB disease. Regarding information resources of TB according to each group of health professionals, physicians primarily obtained TB information from university faculties whereas nurses and other professionals primarily obtained TB information from news media.

### Screening for *M. tuberculosis* infection among participants

We collected data from *M. tuberculosis* screening for a total 299 health professionals at BMH. Among health professionals, the prevalence of positive TST results was highest among nurses (64.1%), with no significant difference (Table 2). A similarly high prevalence of positive results was observed for physicians (56.3%) and nurses (56.2%). The prevalence for health professionals in the emergency department showed the highest frequency of positive TST results among all departments.

**Table 2:** Characteristics of participants in TB-positive and TB-negative groups according to results of tuberculin skin test

	Positive	Negative	*P* value
	*n* = 170	*n* = 129	
Demographics			
Sex-male, n (%)	40 (23.5)	30 (23.3)	1.000^*^
Age- n (%), y			0.978^*^
≤ 29	53 (31.2)	40 (31.0)	
30–39	69 (40.6)	51 (39.5)	
≥ 40	48 (28.2)	38 (29.5)	
Health professionals, n (%)			0.766^*^
Physician	45 (26.5)	35 (27.1)	
Nurse	109 (64.1)	85 (65.9)	
Others	16 (9.4)	9 (7.0)	
Department, n (%)			<0.001^*^
Respiratory Center	16 (9.4)	32 (24.8)	
Emergency	49 (28.8)	22 (17.1)	
Infectious Diseases	16 (9.4)	3 (2.3)	
Nephrology	13 (7.6)	28 (21.7)	
Hematology	36 (21.2)	7 (5.4)	
Out patients	23 (13.5)	19 (14.7)	
Others	17 (10.0)	18 (14.0)	
Years for working experiences as current profession-mean (SD), yr.	9.7 (9.7)	10.3 (7.0)	0.469^‡^
History of TB disease, n (%)			
Personal history (*n* = 292)	0 (0.0)	3 (2.4)	0.077^†^
Family history (*n* = 289)	4 (2.4))	2 (1.6)	0.702^†^
Record of past BCG (*n* = 298)	83 (49.1)	36 (27.9)	<0.001^*^
Record of past TST (*n* = 295)	165 (98.2)	122 (96.1)	0.297^*^
Possibility of exposure with TB patients			<0.001^*^
Yes	100 (58.8)	97 (77.0)	
No	36 (21.2)	4 (3.2)	
Do not know	34 (20.0)	25 (19.8)	

*SD* Standard deviation, *TB* Tuberculosis, *BCG* Bacillus Calmette-Guerin, *TST* Tuberculin skin test

* χ2 test; †Fisher’s exact test; ‡Mann-Whitney U test

### Knowledge about TB among health professionals

The results for each question related to knowledge about TB for each group of health professionals are presented in Table 3. According to our scale, health professionals had high average levels of TB knowledge (overall mean knowledge score = 7.87; SD, 1.31), and physicians had significantly higher scores than nurses and other professionals.

**Table 3:** Clinical and general knowledge of TB according to health professionals

	Physician	Nurse	Others	*P* value
	*n* = 140	*n* = 256	*n* = 44	
Knowledge score, mean (SD)	8.46 (0.96)	7.70 (1.16)	7.64 (1.03)	<0.001
Clinical knowledge about TB-no. (%) of answered				
TB is a treatable disease. (*n* = 435)	139 (99.3)	240 (95.2)	40 (93.0)	0.052
All people with the TB infection develop the TB disease.	128 (93.4)	188 (76.7)	33 (76.7)	<0.001
Symptom that can identify TB (*n* = 427)				
Rate of correct answer-mean (SD), %	92.4 (10.2)	87.9 (10.3)	81.5 (16.2)	<0.001
Risk disease for TB (*n* = 432)				
Rate of correct answer-mean (SD), %	89.7 (14.5)	72.4 (20.7)	69.1 (20.9)	<0.001
Consequences of incomplete or abandoned treatment (*n* = 447)				
Development of drug resistant	135 (96.4)	205 (81.3)	33 (75.0)	<0.001
Spreading infection to other people	102 (72.9)	153 (60.7)	23 (52.3)	0.014
Development of extra-pulmonary or systemic TB	89 (63.6)	132 (52.4)	20 (45.5)	0.040
TB Transmission -answered ‘Yes’-n (%)				
Patients with active TB disease can infect people by coughing. (*n* = 435)	134 (95.7)	244 (97.2)	42 (95.5)	0.671
TB is often spread from person to person through the air. (*n* = 431)	124 (89.2)	229 (92.0)	40 (93.0)	0.631
TB is often spread from person to person through sexual contact. (*n* = 422)	128 (92.8)	224 (92.9)	36 (83.7)	0.122
Patients with active TB disease can infect people by spitting. (*n* = 429)	134 (97.8)	241 (97.2)	44 (100.0)	0.804
HIV-positive patients are more vulnerable to catching TB than HIV-negative. (*n* = 434)	132 (94.3)	230 (92.0)	42 (95.5)	0.663
TB is often spread from person to person by blood. (*n* = 428)	124 (89.9)	230 (93.1)	35 (81.4)	0.041
Patients with active TB disease are more likely to infect others if they have a cough that produces a lot of sputum. (*n* = 433)	132 (94.3)	231 (92.8)	42 (65.5)	0.745
TB can be transmitted through handshakes. (*n* = 431)	136 (97.1)	235 (94.8)	41 (95.3)	0.500
TB can be transmitted through mixing patient’s clothes in the washing-machine. (*n* = 431)	135 (96.4)	233 (94.0)	40 (93.0)	0.557
Global condition of TB in Vietnam				
What is the global condition of TB-burden of Vietnam				<0.001
High-burden country	121 (87.7)	153 (63.5)	25 (56.8)	
Middle-burden country	16 (11.7)	79 (33.6)	14 (35.0)	
Low-burden country	0 (0.0)	3 (1.3)	1 (2.5)	
Respirators relating to TB				
N95 protect healthcare workers and visitors by stopping TB particles from being breathed in - ‘Yes’-n (%). (*n* = 422)	123 (91.1)	215 (88.5)	37 (84.1)	0.658
This is an N95 mask - ‘No’-n (%) (*n* = 410) < picture of gauze mask>	119 (90.8)	213 (93.4)	29 (76.3)	0.004

*SD* Standard deviation, *TB* Tuberculosis, *HIV* Human immunodeficiency virus

Concerning respirators, 9.8% of physicians, 6.6% of nurses, and 23.7% of other health professionals did not know about the N95 respirator and could not recognize it from an image. Regarding the TB burden in Vietnam, 11.7, 34.9, and 37.5% of physicians, nurses, and other professionals, respectively, did not know that Vietnam is a high TB burden country.

### Attitudes of health staff towards TB

Attitudes towards TB for each health professional group are presented in Table 4. The overall mean attitude score was 7.88 (SD, 0.98), and there was no significant difference among groups of health professionals. Although most health professionals considered preventing TB spread in the hospital and identifying new patients with TB to be important, 68.1% of physicians and 62.5% of nurses felt that taking care of these patients was stressful.

**Table 4:** Attitude about TB infection control according to health professionals

	Physician	Nurse	Others	*P* value
	*n* = 140	*n* = 256	*n* = 44	
Attitude score, mean (SD)	7.79 (0.99)	7.93 (0.99)	7.97 (0.88)	0.597
Attitude for works relating to TB				
I am willing to keep my work in the current ward/department? (*n* = 427)	136 (98.6)	242 (98.4)	42 (97.7)	0.730
It is very important to prevent the spread of TB in the hospital? (*n* = 427)	138 (100.0)	243 (98.8)	41 (95.3)	0.040
Do you feel any scarily about the infection from TB patients? – ‘agree,’ n (%) (*n* = 421	123 (91.8)	224 (91.8)	39 (90.7)	1.000
Do you feel any stress for taking care of TB patients? –n (%) (*n* = 429)				0.279
Agree	94 (68.1)	155 (62.5)	32 (74.4)	
Disagree	44 (31.9)	88 (35.5)	11 (25.6)	
Neither agree/disagree	0 (0.0)	5 (2.0)	0 (0.0)	
Finding all of the new case of TB is an important task in controlling the disease. (*n* = 427)	135 (99.3)	241 (97.2)	42 (97.7)	0.487
Agree	135 (99.3)	241 (97.2)	42 (97.7)	
Disagree	1 (0.7)	3 (1.2)	0 (0.0)	
Neither agree/disagree	0 (0.0)	4 (1.6)	1 (2.3)	
It is important to realize more actions to include the community in TB prevention and control. (*n* = 430)				0.046
Agree	137 (99.3)	246 (98.8)	40 (93.0)	
Disagree	0 (0.0)	2 (0.8)	2 (4.7)	
Neither agree/disagree	1 (0.7)	1 (0.4)	1 (2.3)	
The knowledge and awareness of TB in your community is adequate. (*n* = 427)				0.254
Agree	51 (37.0)	86 (35.0)	12 (27.9)	
Disagree	80 (58.0)	138 (56.1)	30 (69.8)	
Neither agree/disagree	7 (5.1)	22 (8.9)	1 (2.3)	
Concerning facility’s barriers for TB infection control				
My ward/department have enough isolation rooms for suspected TB patients. (*n* = 415)				0.076
Agree	16 (11.7)	44 (18.0)	11 (26.2)	
Disagree	117 (85.4)	198 (80.8)	29 (69.0)	
Neither agree/disagree	4 (2.9)	3 (1.2)	2 (4.8)	
I satisfy with the hospital’s TB infection control. (*n* = 369)	36 (27.1)	128 (53.3)	15 (36.6)	< 0.001
Satisfied issues				
Patients’ Isolation room	92 (73.0)	113 (55.7)	22 (55.0)	0.005
Visitor’s rules to hospitalized TB patients	59 (46.8)	75 (36.9)	22 (55.0)	0.048
Windows at patients’ room	60 (47.6)	81 (39.9)	9 (22.5)	0.019
UV lights in place and routinely used	60 (47.6)	99 (48.8)	14 (35.0)	0.278
Fans and opening windows in TB patients’ rooms	46 (36.5)	69 (34.0)	7 (17.5)	0.075
Air filters in TB patients’ rooms				
Routine TB screening for healthcare professionals	64 (50.8)	98 (48.3)	17 (42.5)	0.657
Sputum collecting box/corner for suspected TB patients	37 (29.6)	73 (36.0)	13 (32.5)	0.509
Aeration system of rooms for TB patients and consultation	66 (52.4)	85 (41.9)	12 (30.0)	0.027
Feeling any barriers for implementing TB infection control activities in the hospital				0.043
Agree	60 (44.8)	111 (48.3)	16 (39.0)	
Disagree	67 (50.0)	108 (47.0)	18 (43.9)	
Neither agree/disagree	7 (5.2)	11 (4.8)	7 (17.1)	

*SD* Standard deviation, *TB* Tuberculosis

Concerning feelings about TB infection control in the hospital, only 27.1% of physicians were satisfied with current TB infection control measures, especially in terms of isolation rooms, fans and windows in the rooms of patients with TB, and sputum collection rooms/corners for patients with suspected TB.

### Practices of health professionals regarding TB

The practice scores and responses to each practice question according to health professional groups are presented in Table 5. The overall mean practice score was 7.08 (SD, 1.96), with no significant difference among groups of health professionals. A total 85.0 and 93.4% of physicians and nurses, respectively, wore masks when seeing patients and approximately half of health professionals wore a gauze mask.

**Table 5:** Practices about TB infection control according to health professionals

	Physician	Nurse	Others	*P* value
	*n* = 140	*n* = 256	*n* = 44	
Practice score, mean (SD)	6.89 (2.11)	7.20 (1.83)	6.99 (2.20)	0.531
Wearing mask				
Do you wear mask when you see a patient?	113 (85.0)	225 (93.4)	30 (76.9)	0.002
When you see a patient, what kind of mask do you wear?				<0.001
N95 respirator	23 (16.7)	52 (21.4)	14 (35.0)	
Surgical mask	33 (23.9)	63 (25.9)	4 (10.0)	
Gauze mask	69 (50.0)	123 (50.6)	15 (37.5)	
Never wear mask	13 (9.4)	5 (2.1)	7 (17.5)	
Collecting sputum				
Do you open the window? (*n* = 382)	62 (54.9)	127 (54.7)	22 (59.5)	0.882
What is the barrier for not to open windows?				
Save time	18 (16.8)	28 (14.0)	3 (8.6)	0.479
Do not agree to open windows	6 (5.6)	31 (15.5)	4 (11.4)	0.040
No window	23 (21.5)	50 (25.0)	6 (17.1)	0.544
Windows are fixed and cannot be opened	13 (12.1)	27 (13.5)	8 (22.9)	0.276
No barrier to open windows	21 (19.6)	33 (16.5)	4 (11.4)	0.513
Do you wear mask when you support to collect sputum? (*n* = 399)	117 (95.1)	234 (97.1)	31 (88.6)	0.057
Kind of mask (*n* = 383)				0.003
N95 respirator	29 (22.7)	55 (24.7)	11 (34.4)	
Surgical mask	21 (16.4)	64 (28.7)	5 (15.6)	
Gauze mask	53 (41.4)	82 (36.8)	7 (21.9)	
Treating patients with suspected active TB disease^a^				
Ever treated suspected active TB disease who is treated for other diseases? (*n* = 397)	125 (94.0)	196 (86.0)	28 (77.8)	0.012
What did you do as the first thing?				
Isolate patient	82 (60.7)	109 (46.0)	23 (53.5)	0.023
Offer patient wearing surgical mask	42 (31.1)	80 (33.8)	21 (48.8)	0.097
Wear N95 mask on yourself	47 (34.8)	92 (38.8)	23 (53.5)	0.091
Move patient’s room where airborne infection control can properly apply.	87 (64.4)	152 (64.1)	21 (48.8)	0.146
Educate patients and patients’ relatives for cough hygiene	55 (40.7)	131 (55.3)	23 (53.5)	0.024
Transfer to respiratory center	59 (43.7)	95 (40.1)	29 (67.4)	0.003
Inform about it to your supervisor	30 (22.2)	103 (43.5)	25 (58.1)	<0.001
Do nothing	0 (0.0)	1 (0.4)	1 (2.3)	0.302
Do you inform the importance of cough hygiene to suspected TB patients? (*n* = 408)	118 (88.7)	218 (90.8)	33 (94.3)	0.580
Do you offer wearing surgical mask to suspected TB patients? (*n* = 410)	118 (89.4)	220 (91.3)	35 (94.6)	0.608
Do you move suspected TB patients to the isolation room? (*n* = 390)	84 (66.1)	150 (66.1)	29 (80.6)	0.221
Do you think your ward have enough isolation rooms to accommodate patients suspected of having tuberculosis? (*n* = 407)	17 (13.0)	41 (17.2)	12 (32.4)	0.020

^a^Active TB disease was defined as smear-positive *M. tuberculosis*

*SD* Standard deviation, *TB* Tuberculosis

Most physicians (94.0%) and nurses (86.0%) had ever treated patients with suspected TB. For physicians, the main initial actions when seeing patients with suspected TB included moving the patient to a room with airborne infection control (64.4%), isolating the patient (60.0%), and transferring the patient to the Respiratory Centre (43.7%). For nurses, the primary initial actions were moving the patient to a room with airborne infection control (64.4%), educating the patient and patient’s relatives about TB (55.3%), isolating the patient (46.0%), and informing their supervisor (43.5%). However, only 13.0 and 17.2% of physicians and nurses, respectively, thought that there were a sufficient number of isolation rooms for patients with suspected TB in their wards.

### Factors influencing knowledge scores

Factors influencing knowledge scores associated with TB were estimated using a multiple regression model (Table 6). The analysis revealed that age, a belief that TB is the most important infectious disease, being a medical professional, having previously attended a workshop or seminar, and knowing that Vietnam has a high TB burden were factors associated higher knowledge scores.

**Table 6:** Factors influencing knowledge score associated with TB using multiple regression analysis

	coefficient	SE	*p* value	95% CI
constant	7.307	0.322	<0.001	6.673–7.940
Age	0.014	0.007	0.050	0.000–0.027
Think about TB is the most important infectious disease	0.292	0.110	0.008	0.076–0.509
Health professional	−0.303	0.088	0.001	−0.476- -0.129
Previously attended for workshops or seminars	0.236	0.114	0.038	0.013–0.460
Knew about Vietnam is high TB-burden country	0.673	0.114	<0.001	0.450–0.897

*SE* Standard error, *95% CI* 95% confidence interval, *TB* Tuberculosis

## Discussion

The present study revealed that there are some difficulties with TB infection control measures at a general hospital in Hanoi, including poor understanding self-protection using an N95 respirator and immediate isolation of patients with suspected TB. A high prevalence of latent TB infection among health professionals was observed, in particular those in the emergency department. Providing educational programs and obtaining knowledge from scientific sources among health professionals would help in reducing nosocomial TB infection.

In the health care system of Vietnam, as the central hospital, BMH (the study site) cares for patients who have difficulty with clinical treatment in local hospital settings [16, 17]. Therefore, whereas treatment for patients with TB is provided in TB-specialized hospitals, there is a high likelihood that health professionals in the central hospital see patients who have not yet been diagnosed with TB or who are unaware of having TB but are transported to that hospital. As a result, a high proportion of physicians and nurses at our study site had previously treated patients with TB and suspected TB. Previous autopsy studies in a tertiary referral hospital in other high TB-burden countries also identified a high incidence of TB among patients who were not suspected of having TB [18, 19]; it can be therefore be thought that the occupational risk of TB infection among health professionals may be high. In particular, in the present study, the greatest proportion of TST-positive results was among health professionals in the emergency department. A large number of patients who are transferred from local hospitals and admitted to the emergency department may not be aware of their TB status [20]. This result was compatible with previous studies in South Korea and the United Kingdom [6, 21]. Specific TB control measures, including rapid diagnosis of TB in patients transferred to the emergency department are crucial with the current diagnostic methods, which require substantial time. The finding of an abnormal chest radiograph would be a clue for earlier diagnosis of TB, together with the identification of risk factors and symptoms [21].

In a high TB burden country, appropriate and necessary information of TB is crucial for health professionals in medical facilities, even if they are employed at non-TB-specialized hospitals. In the present study, the main information resource among physicians was university faculties, and that for nurses and other professionals was news media. In addition, the prevalence of physicians, nurses, and other professionals who had ever attended educational programs on TB was low. Although the knowledge scores of each group were high, as health professionals who provide the first diagnosis of TB and provide care for patients with suspected TB, some knowledge gaps were observed, especially in terms of understanding the symptoms and diseases associated with the risk of TB. Delayed diagnosis and treatment of TB has been previously reported in Vietnam [22].

Knowledge about TB transmission is required, as are practices for preventing TB infection, including patient isolation and use of an N95 respirator. These respirators have a high filtration barrier, to protect health professionals from TB infection. N95 respirators are recommended for health professionals caring for patients with TB or suspected TB [8]. However, among participants in the present study, the adherence to N95 respirator use was not observed owing to poor understanding of respirator use. Understanding the importance of respirators by hospital administration for TB infection control and training in the appropriate use of N95 respirators must be included in educational programs for health professionals [23, 24]. Our results of multiple regression analysis indicated that providing proper and comprehensive knowledge regarding TB and TB infection control via workshops or seminars for physicians as well as nurses and other health professionals would help to increase their knowledge. Higher knowledge levels about TB among health professionals and subsequent changes in their attitudes and behaviours would contribute to reducing nosocomial TB infections.

The present study had some limitations. Most measurements relied on self-reporting by health professionals. Although the survey was conducted in an anonymized fashion, the possibility of bias cannot be ignored. The number of participants with TST results was smaller than the number who participated the KAP survey because TSTs were conducted during professionals’ working hours. Therefore, we did not statistically assess the correlation between the results of KAP surveys and the prevalence of latent TB infection. Further investigation is needed among equal numbers of participants with results for both the KAP survey and TST. Although we examined participants for latent TB infection, we were not able to determine whether infection occurred at the hospital or elsewhere. This study was conducted in a single hospital in Hanoi and included health professionals in limited departments. Therefore, the conditions may differ from TB infection control in local settings. Despite these limitations, the present results can contribute to the improvement of TB infection control in general hospitals of Vietnam.

## Conclusions

Although health professionals had high KAP scores, we identified gaps in their knowledge about proper TB infection control, including understanding of self-protection using an N95 respirator and immediate isolation of patients with (suspected) TB. Early awareness of (suspected) TB to prevent transmission, as well as education about obtaining TB knowledge from scientific sources among health professionals, will help in reducing nosocomial TB infection and in implementing proper measures when caring for patients with (suspected) TB. A high prevalence of latent TB infection among health professionals may also suggest the need to strengthen TB infection control, particularly among health professionals in the emergency department.

## Data Availability

Due to restrictions form the study organizers and the ethics committee in the study site, the data cannot be made publicly available. External investigator(s) wishing to access the study data can contact the corresponding author on reasonable request.

## Abbreviations

BCG: Bacillus Calmette–Guerin
BMH: Bach Mai Hospital
HIV: Human immunodeficiency virus
KAP: Knowledge, attitude, and practice
TB: Tuberculosis
TST: Tuberculin skin test
WHO: World Health Organization

## References

[1] World Health Organization. Global tuberculosis report 2018. Available at https://www.who.int/tb/publications/global_report/en/. Accessed 15 Apr 2019

[2] Chu H, Shih CJ, Lee YJ (2014). Risk of tuberculosis among healthcare workers in an intermediate-burden country: a nationwide population study. J Inf Secur.

[3] O’Donnell MR, Jarand J, Loveday M (2010). High incidence of hospital admissions with multidrug-resistant and extensively drug-resistant tuberculosis among south African health care workers. Ann Intern Med.

[4] World Health Organization (WHO). Tuberculosis (TB): latent tuberculosis infection (LTBI). Available at https://www.who.int/tb/areas-of-work/preventive-care/ltbi_faqs/en/. Accessed 15 Apr 2019

[5] Janagond AB, Ganesan V, Vijay Kumar GS, Ramesh A, Anand P, Mariappan M (2017). Screening of health-care workers for latent tuberculosis infection in a tertiary care hospital. Int J Mycobacteriol.

[6] Appleton SC, Connell DW, Singanayagam A, Bradley P, Pan D, Sanderson F (2017). Evaluation of prediagnosis emergency department presentations in patients with active tuberculosis: the role of chest radiography, risk factors and symptoms. BMJ Open Respir Res.

[7] Implementing the WHO Stop TB strategy. A handbook for national TB control programmes. Geneva: World Health Organization. http://whqlibdoc.who.int/publications/2008/9789241546676_eng.pdf. Accessed 15 Apr 2019 26269864

[8] Jensen PA, Lambert LA, Lademarco MF, Ridzon R (2005). CDC. Guidelines for preventing the transmission of *Mycobacterium tuberculosis* in health-care settings, 2005. MMWR Recomm Rep.

[9] Kanjee Z, Catterick K, Moll AP, Amico KR, Friedland GH (2011). Tuberculosis infection control in rural South Africa: survey of knowledge, attitude and practice in hospital staff. J Hosp Infect.

[10] World Health Organization. A guide to developing knowledge, attitude and practice surveys. Geneva. Available at http://apps.who.int/iris/bitstream/10665/43790/1/9789241596176_eng.pdf. Accessed 10 Apr 2019

[11] Noé A, Ribeiro RM, Anselmo R (2017). Knowledge, attitudes and practices regarding tuberculosis care among health workers in southern Mozambique. BMC Pulm Med.

[12] Salame FM, Ferreira MD, Belo MT (2017). Knowledge about tuberculosis transmission and prevention and perceptions of health service utilization among index cases and contacts in Brazil: understanding losses in the latent tuberculosis cascade of care. PLoS One.

[13] Naidoo S, Taylor M, Esterhuizen TM (2011). Changes in healthcare workers’ knowledge about tuberculosis following a tuberculosis training programme. Educ Health (Abingdon).

[14] Chakaya JM, Meme H, Kwamanga D (2005). Planning for PPM-DOTS implementation in urban slums in Kenya: knowledge, attitude and practices of private health care providers in Kibera slum, Nairobi. Int J Tuberc Lung Dis.

[15] Cohn DL, O’Brien RJ, Geiter LJ, Gordin F, Hershfield E, Horsburgh C (2000). ATS/CDC statement committee on latent tuberculosis infection membership targeted tuberculin testing and treatment of latent tuberculosis infection. MMWR Recomm Rep.

[16] Takashima K, Wada K, Tra TT, Smith DR (2017). A review of Vietnam's healthcare reform through the direction of healthcare activities (DOHA). Environ Health Prev Med.

[17] Le D-C, Kubo T, Fujino Y, Pham T-M, Matsuda S (2010). Health care system in Vietnam: current situation and challenges. Asian Pacific J Dis Manag.

[18] Bates M, O'Grady J, Mwaba P, Chilukutu L, Mzyece J, Cheelo B (2012). Evaluation of the burden of unsuspected pulmonary tuberculosis and co-morbidity with non-communicable diseases in sputum producing adult inpatients. PLoS One.

[19] Bates M, Mudenda V, Shibemba A, Kaluwaji J, Tembo J, Kabwe M (2015). Burden of tuberculosis at post mortem in inpatients at a tertiary referral Centre in sub-Saharan Africa: a prospective descriptive autopsy study. Lancet Infect Dis.

[20] Min JY, Kim HJ, Yoon C, Lee K, Yeo M, Min KB (2018). Tuberculosis infection via the emergency department among inpatients in South Korea: a propensity score matched analysis of the National Inpatient Sample. J Hosp Infect.

[21] Fox GJ, Nhung NV, Sy DN, Hoa NLP, Anh LTN, Anh NT (2018). Household-contact investigation for detection of tuberculosis in Vietnam. N Engl J Med.

[22] Huong NT, Vree M, Duong BD, Khanh VT, Loan VT, Co NV (2007). Delays in the diagnosis and treatment of tuberculosis patients in Vietnam: a cross-sectional study. BMC Public Health.

[23] Zelnick JR, Gibbs A, Loveday M, Padayatchi N, O’Donnell MR (2013). Health-care workers’ perspectives on workplace safety, infection control, and drug-resistant tuberculosis in a high-burden HIV setting. J Public Health Policy.

[24] Nichol K, McGeer A, Bigelow P, O’Brien-Pallas L, Scott J, Holness DL (2013). Behind the mask: determinants of nurse’s adherence to facial protective equipment. Am J Infect Control.

